# Combined Liver and Multivisceral Resections

**DOI:** 10.1155/2014/976546

**Published:** 2014-02-11

**Authors:** Martin de Santibañes, Agustin Dietrich, Eduardo de Santibañes

**Affiliations:** ^1^Department of Hepato-Biliary-Pancreatic Surgery & Liver Transplant Unit, Hospital Italiano de Buenos Aires, Perón 4190, 1181 Buenos Aires, Argentina; ^2^Department of General Surgery, Hospital Italiano de Buenos Aires, Perón 4190, 1181 Buenos Aires, Argentina

## Abstract

*Background*. Combined liver and multivisceral resections are infrequent procedures, which demand extensive experience and considerable surgical skills. *Methods*. An electronic search of literature related to this topic published before June 2013 was performed. *Results*. There is limited scientific evidence of the feasibility and clinical outcomes of these complex procedures. The majority of these cases are simultaneous resections of colorectal tumors with liver metastases. Combined liver and multivisceral resections can be performed with acceptable postoperative morbidity and mortality rates only in carefully selected patients. *Conclusion*. Lack of experience in these aggressive surgeries justifies a careful selection of patients, considering their comorbidities.

## 1. Introduction

Multivisceral resections associated with liver surgery are infrequent procedures that require considerable skills and extensive experience in general and liver surgery. Information regarding the feasibility and clinical outcomes of these combined procedures is very limited. The majority of cases come from colorectal carcinoma with synchronic liver metastases [1].

Over the last decade, there has been substantial progress in the understanding of liver anatomy and the technical aspects of major resections. Anaesthetic management and perioperative and intensive care have also significantly improved, making this kind of extensive surgeries feasible. However, it is important to carefully select the patients who will benefit from these major procedures. Properly defined selection criteria to do so are missing. The aim of this paper is to review the scientific evidence related to these complex procedures.

## 2. Surgical Indications for Combined Liver and Multivisceral Resections

Combined liver and multivisceral resections can arise in the case of an en bloc resection of tumors that have directly infiltrated other organs or in the circumstance of simultaneous resections of primary tumors along with distinct sites of metastatic extent. The last scenario has the advantage of offering a staged procedure, therefore avoiding a simultaneous multivisceral resection and its related risk.  Table 1 shows the pathological etiologies that can be part of these clinical scenarios. The lack of experience in these aggressive approaches justifies a careful selection of patients, considering their comorbidity and procedure related complications rate.

## 3. Importance of Imaging in Patients' Selection 

**Figure 1:**
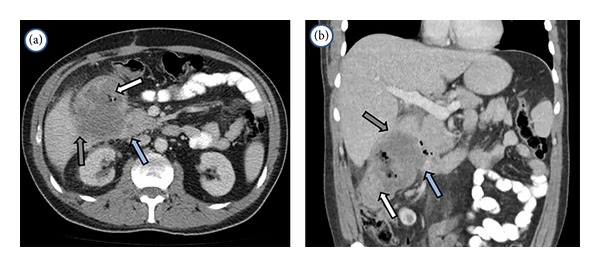
Abdominal and pelvic multidetector computed tomography (MDCT) in a patient with a large colonic tumor (white arrow), which compromises duodenum and pancreatic head (blue arrow) and the right liver (grey arrow).

**Figure 2:**
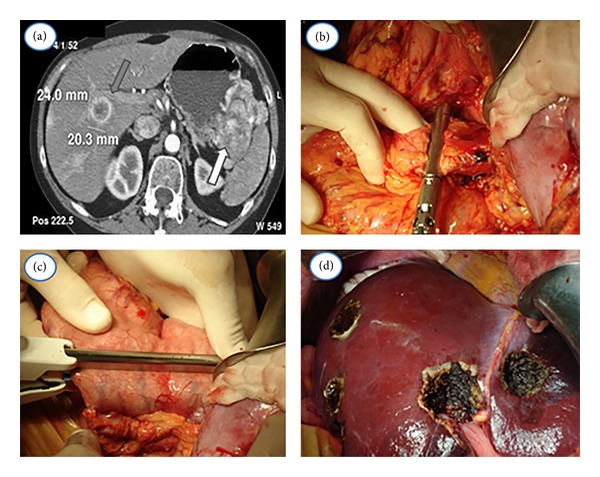
MCDT (a) and intraoperative images (b, c, and d) of a patient with an advanced pancreatic neuroendocrine tumour. (a) MCDT of a patient with diagnosis of a pancreatic neuroendocrine tumor, which involves the pancreas, splenic hilus, and the stomach (white arrow) with liver metastases (Grey arrow). (b) Distal pancreatectomy and splenectomy. (c) Atypical gastrectomy. (d) Multiple liver metastasectomies.

A radiological assessment enables a successful and effective stratification of patients that could result in better surgery outcomes. Multidetectors computed tomography (MDCT) and magnetic resonance imaging (MRI) have the advantage of allowing for preoperative staging and planning a surgical strategy. Both can evaluate the relationship between the primary tumor and the adjacent structures (stomach, colon, kidney, etc.), therefore anticipating multivisceral resections [2, 3] (Figures 1 and 2). They can also determine venous commitment (inferior vena cava, renal veins, superior mesenteric vein, and portal vein) or arterial invasion (celiac axis, hepatic artery, superior mesenteric artery, and aorta) to select candidates for neoadjuvant treatment [4].

They are valuable as well for the:

- assessment of liver tumor load (size, number of lesions, etc.) and evaluation of peritoneal and extrahepatic disease [5, 6],
- determination of liver volumes and estimation of hypertrophy degree of the future liver remnant (FLR) [2, 7, 8],
- surgical planning of hepatic resection centered on the anatomical relationship of the tumor (vascular structures) [2] and radiological staging to increase the rate of resection with curative purpose [2, 9],
- evaluation of biological tumour response to neoadjuvant/adjuvant chemotherapy [10],
- patients' followup after surgical resection.

Depending on the origin of the primary tumor (colorectal, neuroendocrine, etc.), it is important to exclude the presence of distant metastases (brain, lung, and bones) using other imaging methods such as positron emission tomography scan, Octreoscan, and bone scintigraphy.

## 4. Preoperative Stratification of Patient's Surgical Risk 

### 4.1. Major Abdominal Surgery

Study of preoperative risk factors in major abdominal surgery can improve patient selection and postoperative outcomes. Borja-Cacho et al. [11] assessed the factors collected by the American College of Surgeons National Surgery Quality Improvement Program (ACS NSQIP) in major cancer surgeries to predict adverse operative events. This multicentric study highlights that older age (≥75 years) and ASA score (>3) can predict prolonged length of stay, major complications, and 30-day mortality. Cardiac and pulmonary diseases also present direct relationship with patients' postoperative outcomes in major abdominal surgeries [12]. This is caused by inadequate tissue oxygenation during the perioperative course because of a cardiorespiratory malfunction [13]. Al-Refaie et al. [12] found similar results in the ACS NSQIP, reporting a higher incidence of higher operative mortality, greater frequency of major complications, and more prolonged hospital stays in older patients (>75 years).

In patients who underwent major abdominal surgeries, preoperative anesthetics evaluation becomes indispensable. The American Society of Anesthesiologists (ASA) physical status classification is the most prevalent and worldwide used score that stratifies patients according to their preoperative risk. Others describe ASA score as a strong predictor of hospital length stay [14]. There still remains the need to identify more preoperative risk assessment tools in order to predict with higher accuracy the incidence of postoperative complications [15].

### 4.2. Liver Resections

Schroeder et al. [16] analysed records of the National Surgical Quality Improvement in USA of postoperative morbidity and mortality in 587 patients who underwent liver resection, highlighting ASA score as a superior score than other indexes to predict postoperative morbidity. With respect to this, Belghiti et al. [17] among 747 hepatectomies (45% major liver resections) demonstrated that the ASA score not only was an independent risk factor for postoperative complications, but also significantly influenced patient mortality.

Obesity and diabetes are known related factors associated with hepatic steatosis and steatohepatitis [18], both associated with adverse outcomes after liver resection [19]. This population of patients also present a higher risk of anastomotic leaks, especially when undergoing rectal resections [20]. The use of body mass index (BMI) as a measure of patient obesity and as predictor of postoperative morbidity is clearly discussed. For ACS NSQIP, BMI has minimal association with short-term operative outcomes after major cancer surgery [21].

Specifically, even though neoadjuvant treatment appears to be well tolerated and often successful, some reports have informed that chemotherapeutic agents are associated with significant hepatotoxicity and resulting liver failure [22]. The histopathologic changes described in liver specimens include steatosis [23], sinusoidal injuries [24], and steatohepatitis [25]. These histologic changes may generate higher morbidity and even postoperative mortality.

### 4.3. Combined Resections

There is a critical difference in morbidity and mortality when simultaneous nonhepatic procedures were associated with major liver resections [26–28]. Although the lack of evidence in combined resections, prognosis risk factors like ASA score, associated pancreas and liver resections on elderly patients should be highlighted [11]. Additionally, the surgeons' experience and performance at high-volume hospitals are other complimentary factors. Birkmeyer et al. [29] analysed the relation between volume and outcomes in 2.5 millions major surgical procedures in USA, finding large differences in mortality between very-low-volume and very-high-volume hospitals. Perioperative outcomes of major pancreatic or hepatic resections present a solid relationship with the number of performed procedures at a particular hospital [30–33]. Surgeon's volume seems to be an independent prognostic factor of patient's postoperative outcomes [34]. High-volume surgeons could improve postoperative outcomes after pancreatic or hepatic surgery [35, 36].

## 5. Intraoperative Risk Factors

Longer operation time, blood loss, and more frequent blood transfusions are commonly observed in multivisceral resections [37]. Operative time longer than 300 minutes due to pancreatic malignancies was identified as an independent risk factor for the development of intra-abdominal complications [38] with a higher risk of septic events [39].

Blood loss remains a critical aspect during liver resection. Excessive bleeding and subsequent transfusions correlate with postoperative morbidity [40]. Jarnagin et al. [41] described blood loss as an independent predictor of perioperative morbidity and mortality in patients who underwent major liver resection associated with another surgical procedure. In the last years, intraoperative fluid management was reviewed [42, 43]. Adequate anaesthetic techniques to preserve a low central venous pressure, less than 5 mmhg, during extrahepatic dissection and parenchymal transection became necessary to minimize bleeding and the need of blood transfusion [44]. Monitoring lactate levels during the operative time represent an important parameter to control fluid administration [45].

## 6. Postoperative Patient Management

We already discussed the importance of high-volume centers in surgical outcomes. Dimick et al. [46] analyse the frequency of rounds by an intensivist in ICU of 35 hospitals, associating daily rounds with shorter lengths of stay and decreased frequency of postoperative complications. Another study evaluated the relationship between nurse-to-patient ratios in the ICU, showing that fewer nurses per patient increase number of postoperative respiratory related complications [47]. Linke et al. [48] highlighted the role of the surgeon as a leader of a multidisciplinary team in the ICU, not only for his unique knowledge of the patient's anatomy and physiology, but also to make a rapid diagnosis of surgical related complications.

Postoperative thrombosis is another critical issue in these patients. The routine use of venous thromboembolism chemoprophylaxis after hepatic surgery remains controversial, especially in complex resections, when the risk of postoperative bleeding complications influences bleeding more than the risk of postoperative thrombosis [49, 50]. However, due the short experience in liver resections associated with other abdominal procedures, indications of perioperative chemoprophylaxis should be evaluated individually.

## 7. Surgical Strategies

### 7.1. The Role of Staging Laparoscopy

Staging laparoscopy is a simple and minimally invasive method to recognize occult distant metastatic disease and prevent nontherapeutic laparotomies. However, during the last decade the ability of preoperative imaging to identify metastatic (<5 mm nodules) and locally advanced tumors (vascular invasion) has questioned the role of staging laparoscopy [51]. This method could be indicated in certain situations such as histological confirmation of peritoneal nodules, or high levels of tumor markers.

### 7.2. Abdominal Exploration and Oncological Surgical Principles

Extended midline incision or a bilateral subcostal incision with midline extension allows adequate access to the upper, lower, or both abdominal contents, depending on the location of the neoplasm. Vertical midline incisions can be combined with transverse laparotomies. Systematic exploration of the entire abdominal cavity is mandatory, in order to rule out unexpected tumor extension.

It is essential not to make irreversible manoeuvres without prior security of primary tumor resectability, especially if there is preoperative suspicion of vascular invasion. The “artery first approach” of the superior mesenteric artery may be helpful for this purpose, particularly in pancreatic malignancies [52].

Multivisceral resections follow the general principle of all oncological surgery attempting an en bloc tumor resection: to achieve clear margins a border of healthy tissue has to be included in the resection. If there are doubts with tumor-free margins, it is essential to make frozen biopsies. Other criteria include systematic lymphadenectomy. The extension of lymph node resections will depend on the origin of the primary tumor.

### 7.3. Liver Approach

Intraoperative liver ultrasound (IOLUS) represents an essential component of modern liver surgery. It has the potential to show preoperative undetected liver metastases in up to 10–20% of patients [53]. The IOLUS permits the evaluation of hepatic vascular anatomy and its relationship with tumor lesions, supervising the level of resection and the potential for resectability [54].

Associated vascular control techniques in liver surgery such as portal triad clamping and total hepatic vascular exclusion emerge as strategies to perform a safe liver resection minimizing blood loss, controlling hepatic inflow, outflow, or both [55, 56].

Commonly, major liver resections are mandatory to reach tumor-free surgical margins [57]. However, extended hepatectomy increases the risk of the development of postoperative liver failure (PLF) and has been shown to be a predominant cause of hepatectomy related mortality [58].

The assessed FLR volume to avoid PLF should be at least 20% of total liver volume in healthy livers and 30–40% in diseased livers [59, 60]. Portal vein occlusion represents the gold standard technique to induce liver hypertrophy of the FLR, allowing a safe preservation of hepatic reserve to decrease the incidence of PLF [61].

### 7.4. Simultaneous Resections

Due to lack of evidence of combined liver and multivisceral resections, surgical approaches in these scenarios remain controversial.

The largest experience comes from colorectal metastases. Many surgeons support a simultaneous approach as a safe treatment to liver metastases due to colorectal cancer [62–64]. Weber et al. [62] in his series of 97 patients with synchronous colorectal liver metastases divided patients treated with simultaneous approach and those who underwent a delayed resection, showing that morbidity and mortality rates were similar in both groups. In the same line, de Santibañes et al. [63] presented 185 consecutive patients who underwent simultaneous colorectal and hepatic resection for colorectal malignancy with low rates of postoperative morbidity and mortality (20.5% and 1.08%, resp.). Viganò et al. [64] showed similar results regarding morbidity and mortality. Maybe the most controversial factor and predictor of postoperative poor prognostic is the need to perform an extended hepatectomy. Reddy et al. [65] found an increased mortality and severe morbidity compared to minor hepatectomy in his series of 610 patients who underwent simultaneous or staged resections.

Locally advanced gastric carcinoma can be also associated with multivisceral resection, with acceptable perioperative morbidity, mortality (1.9–15%), and 5-year survival (0–40%) [66]. A study showed that the most common combined resected organs were the spleen, pancreas, transverse colon, and liver and were not found to be predictors of poor survival on multivariate analysis [67].

Combined liver and multivisceral resections are infrequent procedures, which demand extensive experience and considerable surgical skills. The lack of experience in these aggressive surgeries justifies a careful selection of patients, considering their comorbidities and should be performed in high-volume centers.

## Figures and Tables

**Table 1:** Etiology for combined liver and multivisceral resections.

Primary tumors with liver infiltration	
Retroperitoneal sarcomas	
Renal tumor	
Adrenal tumor with liver and/or vena cava	
Tumors with splanchnic origin	
Metastatic tumors	
Colorectal cancer	
Noncolorectal nonneuroendocrine metastases	
Neuroendocrine tumor	
Gist tumors	
Liver tumor with splacnic infiltration	
Hepatobiliary tumor that invade splacnic organs: hepatocarcinoma, cholangiocarcinoma, gallbladder carcinoma, hepatic sarcomas, and other mesenchymal tumors	
